# Boltzmann equations and ab initio calculations: comparative study of cubic and wurtzite CdSe

**DOI:** 10.1186/s40064-015-1321-z

**Published:** 2015-09-24

**Authors:** A. Abbassi, Z. Zarhri, Ch. Azahaf, H. Ez-Zahraouy, A. Benyoussef

**Affiliations:** Laboratory of Magnetism and High Energy Physics (URAC 12), Faculty of Sciences, Mohammed V University, B.P. 1014, Rabat, Morocco

**Keywords:** DFT, Optical properties, CdSe, Wien2K, Boltzmann, Transmittance, Optical absorption, Band structure, Optoelectronic

## Abstract

The electronic and optical properties of CdSe in two phases, cubic and wurtzite, have been studied by first principal calculations using the density functional theory. The optical parameters such as transmittance, optical absorption, refractive index and extinction coefficient have been investigated. We have calculated also the band structure, and total/partial density of state using the full potential-linearized augmented plane wave method with the local density approximation, generalized gradient approximation and the modified Becke–Johnson functional (mBJ), implemented in the Wien2k package. With the mBJ approximation the gap found for cubic and wurtzite structure is direct and is equal to 1.85 and 1.7 eV respectively, what corresponds to the experiment results. The optical absorption is significant in the ultraviolet field while it becomes low beyond 600 nm in the visible light for CdSe in different structures. From λ = 400 nm the transmittance is stable and reaches 80 %. With Boltztrap package, we have investigated also that with increasing temperature, the electrical conductivity increases. During the calculation, the cubic structure has presented an isotropy. While for wurtzite CdSe, the propagation of waves into system is different in xx and zz directions. These results can be exploited in several applications of CdSe in optoelectronic devices.

## Background

The semiconductor CdSe is type II–VI, consisting of an element of column II in the periodic table and an element from column VI. The cadmium atom is type II, it has two electrons in the valence band in orbital s, cd (4d10 5s2). The Selenium atom is material of type VI, it has six valence electrons in the s and p orbitals: 3d104s24p4. The valence band of the massive CdSe crystal consists essentially of the selenium p orbitals and the conduction band, of the cadmium orbital s. The massive CdSe material exists in two structures, cubic and wurtzite. The unit cell of Cd and Se is a hexagonal packing. The other form is the cubic structure, where the unit cell is cubic face-centered.

This type of semiconductors is considered an excellent candidate in optical devices technology, optical memories with high-density, transparent conductors, laser devices (Klude et al. 2002), photodetectors (Matsumura et al. 2002; Uthana and Reddy 1981) photoelectric sensors (Fuentes-Hernandez et al. 2004), solar cells (Merad et al. 2005; Merad et al. 2006; Karl and Jr 1982; Murray et al. 1993). It can be also used as a biological labeling (Jr et al. 1998), and in spintronic devices (Beaulac et al. 2008). The CdSe is transparent in infrared radiation, it is used sometimes in the fabrication of photoresistors and in thin layers for instruments using infrared light. This material is also highly luminescent.

CdSe crystallizes in two structures.

A hexagonal one, with space group P63 mc and the lattice parameters a = b = 4.299 A° and c = 7.010 A°. A cubic one, with space group F-43m and a = b = c 6.05 A°.

Several studies and research were treated CdSe using WIEN2k, treating its electronic properties, gap band (Akinci et al. 2009). The aim of this work is to investigate the electronic (DOS, PDOS, band structure), optical (absorption, transmittance, extinction factor, refractive index) and electrical (electrical conductivity) properties in both, CdSe Cubic and wurtzite phases. We will show that we can use CdSe in several applications of opto-electronic and photovoltaic.

This study is also a comparison between the two phases, we will demonstrate that our results obtained with this approximation mbj are in agreement with the experimental data.

## Calculation method

The present calculation is based on ab initio electronic structure. The first principles DFT calculations have been made with the full potential linearized augmented plane wave (FP LAPW) method using the WIEN2k program (Blaha et al. 2011). We also have to mention that our calculations have been performed using different approximations GGA (Perdew et al. 1996), LDA (Slater 1951) and modified Becke- Johnson functional (Blaha and Schwarz 2006).

In order to define the calculation of wave function, we used an energy cutoff of −8.0 Ryd for the LAPW. In this calculation we work with the 500 K-point and the self-consistent was found stable and converge at 10^−6^ Ryd.

The dielectric function is given by the following expression (Abbassi et al. 2015):

1 $$\varepsilon \left( \omega \right) = \varepsilon_{1} \left( \omega \right) + i\varepsilon_{2} \left( \omega \right) = N^{2}$$

with

2 $$N\left( \omega \right) = n\left( \omega \right) + ik\left( \omega \right)$$

The dielectric function comprises two terms: real and imaginary, these two terms describe the variation of optical parameters, the imaginary part of this function is related to the optical conductivity by the following equation:

3 $$\varepsilon_{2} = \frac{\omega \sigma }{{\varepsilon_{0} [\tau_{p}^{2} \left( {\omega_{0}^{2} - \omega^{2} } \right)^{2} + \omega^{2} ]}}$$

The real part $\varepsilon_{1} \left( \omega \right)$ allows also the calculation of other optical parameters, namely, the refractive index and extinction coefficient or attenuation K-factor, which describes the energy loss of a radiation passing through the materials, this relation is given as follows:

4 $$\varepsilon_{1} = n^{2} - k^{2}$$

It is also known that the absorption coefficient is related to the extinction coefficient what can be described by the following relation:

5 $$\alpha = 2\pi k/\lambda$$

To investigate the transport properties of CdSe, we exploit the structure band data calculated with Ab initio method and fitted into Boltzmann package, using the Boltzmann theory and the approach of rigid band (Madsen and Singh 2006; Ziman 2001). We can learn from these approaches that the conductivity that depends on transport distribution can be given by:

6 $$\sigma_{\alpha \beta } \left( \varepsilon \right) = \frac{1}{N}\mathop \sum \limits_{i,k} \sigma_{\alpha \beta } \left( {i,k} \right)\frac{{\delta \left( {\varepsilon - \varepsilon_{i,k} } \right)}}{\delta \left( \varepsilon \right)}$$

We have noticed that α and β are the tensor indices, *ɛ*_*i*,*k*_ is the band structure. N denotes the number of k-points that are sampled in the BZ.

## Electronic properties

### Band structure, total and partial DOS

**Fig. 1:**
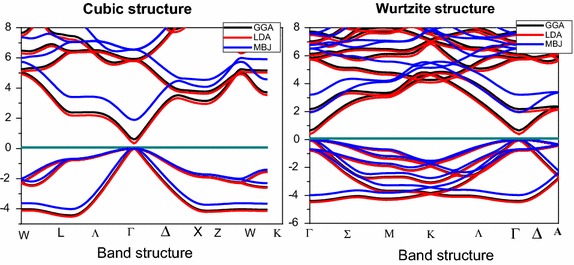
Band structure of cubic and wurtzite CdSe

Figure 1 shows the band structures of CdSe in different Cubic and hexagonal structures. This study was realized with different GGA, LDA and mBJ approximation. It is shown that the gap is direct in Г point for the cubic and hexagonal structures. These different approximations give different gap values. Therefore the gap value with the mBJ (modified beak-Johnson) is close to the value of the experimental data (Kale and Lokhande 2005). The potential of semi local exchange, which retrieves the local density approximation (LDA) and GGA to a constant electron density, leads to the calculations that are more accurate. Table 1 shows the different values of the gap obtained.

**Table 1:** The different values of the gap energy

	LDA (eV)	GGA (eV)	mBJ (eV)	Experiment
Gap–cubic phase	0.412	0.72	1.85	2.3 Kale and Lokhande (2005)
Gap-Wurtzite phase	0.367	0.634	1.7	1.7 Kale and Lokhande (2005)

**Fig. 2:**
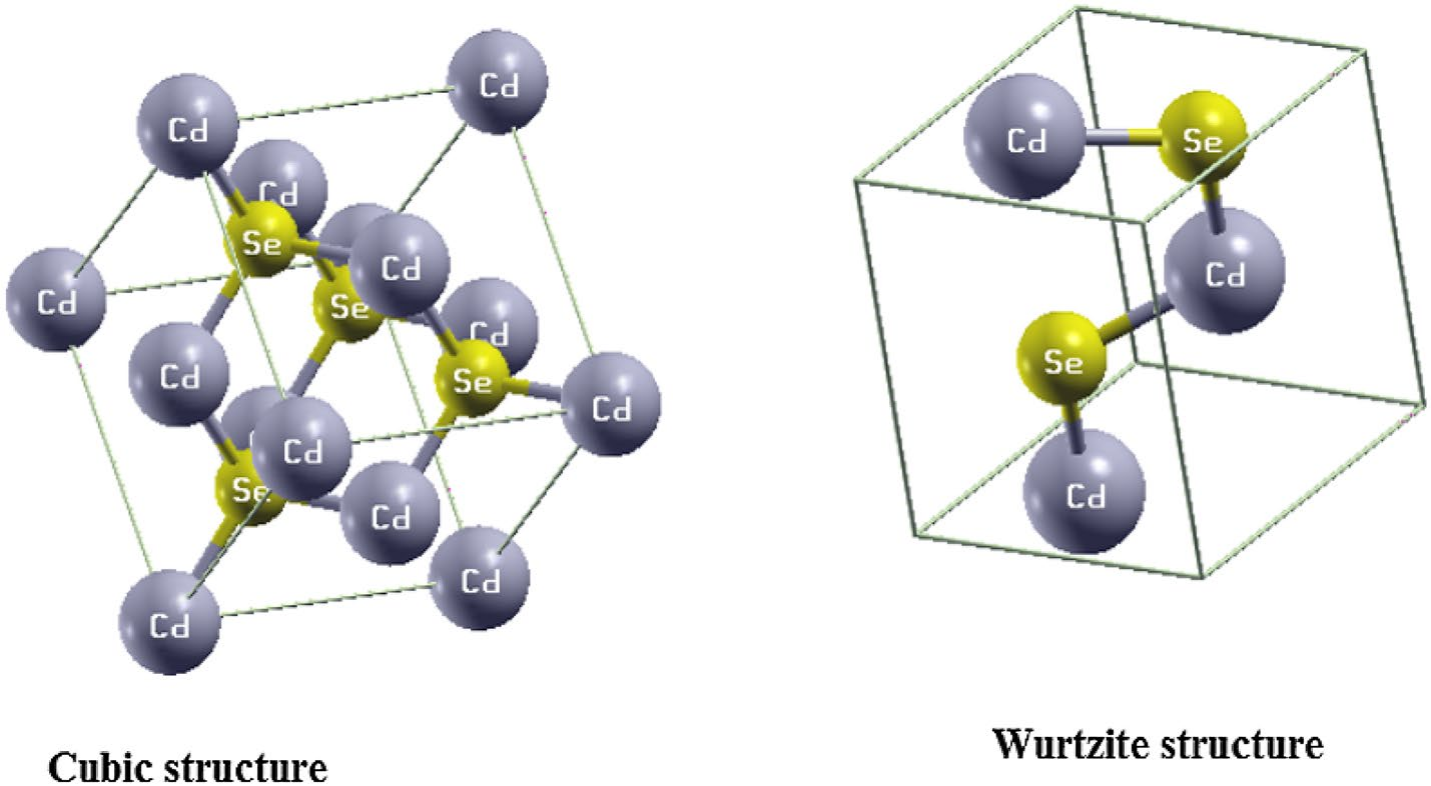
CdSe structures, cubic and wurtzite

We have noted that both structures present DOS with presence of different regions of bands and energy levels. In the case of the cubic structure we observe that a very intense region appears in the conduction band and especially from 4.5 eV. The hexagonal structure as shown in Fig. 2, presents a region with a very intense energy levels in valence and the conduction band. We will make a detailed calculation in the total and partial density of states in order to understand this electronic structure.

**Fig. 3:**
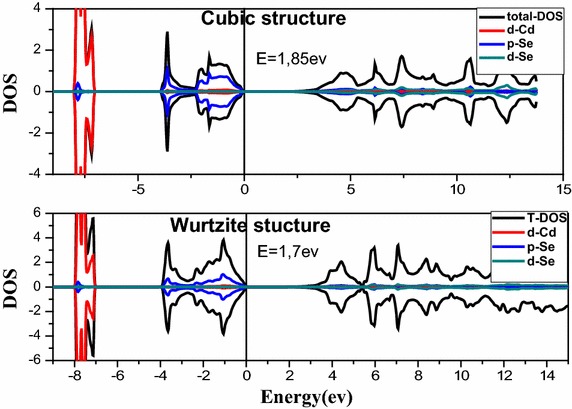
Total and partial density of state

Figure 3 shows the total and partial density of state of CdSe in different structures. For the cubic structure it has several regions where energy level appears. In the valence band we can note that an intense band appear from −3.89 to 0 eV mainly due to the orbital p of Se and another range from −8.04 to −7.09 eV mainly due to the orbital d of Cd. The conduction band also has an intense range beyond 1.85 eV mainly due to the orbital d of Se and low occurrence of p and d of Se.

In the hexagonal structure, the valence band is formed of an intense region from −3.89 to 0 eV essentially due to the orbital p of Se and a region from −8.04 to −7.04 eV which is mainly due to the orbital d of Cd. Beyond 1.7 eV, the conduction band is formed with a low occurrence of orbital d of Cd and p, d of Se.

It is also noted that the difference between the gap energy of wurtzite and cubic structure is ΔEg = 0.15 eV.

### Optical and transport properties

**Fig. 4:**
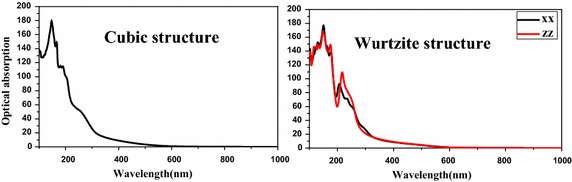
Optical absorption of CdSe in different structures

Figure 4 shows the variation of absorption as a function of a wavelength for CdSe in different structures. This study shows that CdSe in the cubic structure is isotropic. The propagation of radiation hν in xx and zz directions is the same in the system. It has a significant absorption in the ultraviolet range and becomes very low from 600 nm. In the range of 380–600 nm in the visible light, CdSe has a low absorption and substantially lower than the ultraviolet.

CdSe in wurtzite structure is not completely isotropic, the propagation of radiation in the direction xx and zz is different, especially in the range of 200–300 nm. The variation of the absorption in the region of ultraviolet and visible light of Wurtzite does not present a large difference with the cubic structure.

**Fig. 5:**
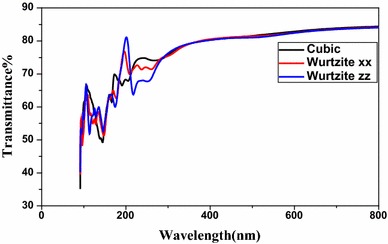
Transmittance of CdSe in different structures

According to the Fig. 5, the transmittance of CdSe is stable and can reach 80 % from λ = 400 nm for the cubic and hexagonal structures. The hexagonal system is not isotropic, it does not present the same variation of transmittance, particularly in the ultraviolet field.

In the ultraviolet field, the transmittance is not stable and shows the lowest value for the cubic structure at λ = 144 nm where the reached value amounts 40 %. CdSe in its structure behaves as transparent in the visible light and absorbent in ultraviolet. These optical properties can be exploited in various optical applications.

The refractive index is also one of the optical properties of the systems, it describes the behavior of radiation propagating in materials. We have noted that in the absorbent systems, the refractive index consists of two expressions, real and imaginary parts as shown in Eq. (2).

It is also noted that the imaginary part of the refractive index shows the attenuation K of the wave, which is essentially designed to investigate the energy loss of the radiation. It is related to the absorption coefficient by the Eq. (5).

**Fig. 6:**
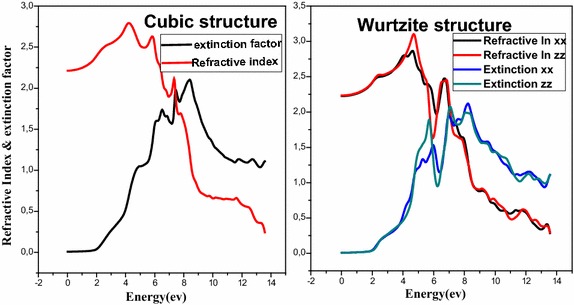
Refractive index and extinction coefficient of CdSe in different phases

**Fig. 7:**
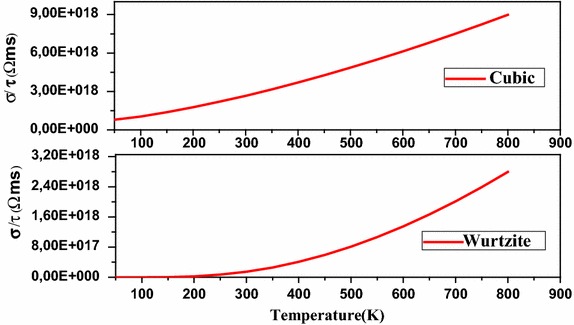
The electrical conductivity versus temperature of CdSe in different structures

Figure 6 shows the variation of the refractive index n and the extinction coefficient K as a function of energy. The curves show that for the energies lower than 4 eV, CdSe is transparent, the highest values reached of the refractive index are: 2.78 for the cubic structure what corresponds to 4.2 eV energy, and for the hexagonal structure the n = 3.06 what corresponds to the 4.68 eV energy. The refractive index decreases in both structures with increasing of energy which is in agreement with these experiment results (Ninomiya and Adachi 1995). Regarding to the extinction coefficient, it increases to a value of 2.09 what corresponds to E = 8.36 eV for cubic structure and to K = 2.11 what correspond to 8.12 eV in wurtzite structure (Fig. 7).

Using the Boltzmann equation mentioned above, we have calculated the variation of the electrical conductivity as a function of temperature. This calculation is very important to describe the electrical behavior of CdSe,

It has been observed that CdSe in cubic and wurtzite structure has an increasing electrical conductivity while the temperature increases, which means that the number of free electrons increases. Our results are in agreement with the experimental results (Suresh and Arunseshan 2014). This conductivity is mainly due to the presence of selenium vacancies as is reported by several researchers. Therefore, we can conclude that CdSe can be exploited as a semiconductor in photovoltaic applications, the conductivity and transmittance which reaches 80 % are significant parameters that allow us to consider this material as a candidate in solar cell applications.

## Conclusion

We have calculated the electronic, optical and electrical properties of CdSe in different phases, cubic and wurtzite model with different approximations. Using Boltztrap equations and FP-LAPW method with the generalized gradient approximation (GGA), local density approximation and mBJ approach. The calculations show that the cubic structure is isotope while for wurtzite structure the propagation of radiation hν is different in xx and zz directions.

The decreases of absorption coefficient in the visible light, a stable transmittance which reach 80 % in the visible light range beyond 400 nm were showed. We have investigated also that with increasing temperature, the electrical conductivity increases. The optical absorption is significant in the ultraviolet field. We have shown favorable results between calculations and the existing experimental data, these results confirm the quality that can present CdSe to be used as suitable material in optoelectronic and photovoltaic applications.
